# The impact of network topological structures on systematic technology adoption and carbon emission reduction

**DOI:** 10.1038/s41598-021-99835-3

**Published:** 2021-10-14

**Authors:** Huayi Chen, Huai-Long Shi

**Affiliations:** 1 College of Economics and Management, Nanjing University of Aeronautics and Astronautics, 29 Jiangjun Avenue, Nanjing, 211106 China; 2 School of Management Science and Engineering, Nanjing University of Information Science and Technology, Nanjing, 210044 China

**Keywords:** Computational science, Environmental impact, Energy economics, Energy policy

## Abstract

This paper investigates how the topological structure of the technological spillover network among agents affects the adoption of a new clean technology and the reduction of system’s carbon emissions. Through building a systematic technology adoption model with technological spillover effect among agents from the network perspective, this paper first illustrates how the new technology diffuses from the earlier adopters to the later adopters under different network topological structures. Further, this paper examines how the carbon emission constraints imposed on pilot agents affect the carbon emissions of other agents and the entire system under different network topological structures. Simulation results of our study suggest that, (1) different topological structures of the technological spillover network have great influence on the adoption and diffusion of a new advanced technology; (2) imposing carbon emission constraints on pilot agents can reduce carbon emissions of other agents and thereby the entire system. However, the effectiveness of the carbon emission constraints is also largely determined by the network topological structures. Our study implies that the empirical research of the network topological structure among the participating entities is a pre-requisite to evaluate the real effectiveness of a carbon emission reduction policy from the system perspective.

## Introduction

Historical evidence and economic theory suggest that, advances in technological knowledge are the single most important contributing factor to long-term productivity and economic growth^1^. Without technological change, especially in energy technologies, it will be difficult to deal with the dynamics of climate change, and its impacts on ecosystems and economic systems^2^.

Generally speaking, there are two ways for an entity to achieve technological progress: the first way is to enhance the technological innovation capabilities endogenously, and the second way is to acquire advanced technology via the diffusion from other entities^3^. Most extant studies have focused on the internal drivers of the technological change. For example, technological learning is acknowledged as an endogenous driving force of technology adoption^4–6^. As for the externalities, such as the technological spillover effect, it has also gained its popularity recently^1,7–9^. Technological spillover effect across different entities constitutes an important mechanism for a new technology’s adoption and diffusion globally. These spillovers happen in the format of, e.g., flows of knowledge, goods or talents^7^.

The spillover among entities can be naturally captured by the network theory. Literature on spillover process from the network perspective is abundant. The majority of these studies related to our research in this paper, such as the technological spillover, knowledge spillover, or learning spillover, are empirical studies. They conduct investigations to identify the network structures and properties with real data. For example, the study conducted by Barreto and Klaassen examines the possibility that technology learning accumulated in a given region may spillover to other regions^10^. Singh examines whether interpersonal networks help explain patterns of knowledge diffusion using the data of patent citation to measure knowledge flows^11^. Fershtman and Gandal construct a two-mode network and demonstrate that the structure of these networks is associated with project success, which suggests the existence of direct and indirect project knowledge spillovers^12^. Most recently, Wang et al. examine main semiconductor companies’ network structures and channels of knowledge spillover with patent bibliometrics and social network analysis^13^. Ji et al. combine the connectedness network framework and the ensemble empirical mode decomposition method to investigate the dynamic information spillovers among the returns of crude oil, refinery product and natural gas^14^.

Existing studies that integrate complex networks with modeling and analyzing technology adoptions are mostly from individual, household or firm levels. For example, Sykes et al. argue that an individual’s social network characteristics can aid the understanding of new information system use and enhance the understanding of technology use^15^. Peng and Mu propose a framework to examine how social network dynamics affect online technology adoption^16^. Peng and Dey propose a dynamic view of technology adoption by considering network centrality and closure and the content of potential information flows within a network^17^. Zaffar et al. conduct a series of experiments with agent-based modeling technique to identify strategically located firms that can influence the software diffusion process significantly in a network^18^. In the field of energy technologies, Bale et al. build a model based on network with dynamic nodes representing households who choose whether or not to adopt a new energy technology^19^. They highlight the value of social networks in the models of energy decision-making and policy interventions. Du et al. show the impact of interactions between individuals in social network to the mass rollout of energy efficiency technologies^20^. Vega and Mandel propose a methodology to infer the network structure of technology diffusion among countries from adoption data and apply it to wind energy technologies^21^. They also use the inferred network to characterize strategies in order to maximize the spread of new technologies. Li et al. employ a complex network evolutionary game method to investigate the dynamics of policy impacts on electrical vehicle diffusion in different scale networks^22^.

Given the above, our work contributes to the relevant literature from the following aspects: first, existing literature rarely explores technology adoption with multiple interacting decision agents from the system perspective. Systematic technology adoption models are normally built for social planners. The purpose of these models is commonly to meet a certain system objective while satisfying different system constraints^9,23–25^. Studies on modeling systematic technology adoption commonly assume one global decision maker, and ignore the existence of multiple decision makers in the entire system who make decisions independently and also have interactions with each other. Hence, this paper intends to investigate the systematic technology adoption through combining both ‘internal factors’ and ‘external factors’, i.e., endogenous technological change and technological spillover effect among multiple agents^26^. Second, and more importantly, existing literature rarely explores the influence of the technological spillover network structure among agents. When there are many agents exist in the entire system, each one of them is “selfish” and only cares about optimizing the technology adoption strategies for a portion of the entire system it represents. The technological spillover process among agents proceeds over a structure^27^, which can be determined by the network topological properties. Different network topological structures provide distinct channels for the later adopters to acquire the knowledge and experience of using a new technology from the earlier adopters at different paces. As a consequence, the adoption of the new technology and the corresponding carbon emissions in the entire system should also be determined by the network topological structures.

With the discussion above, here we present the research questions of this paper:

- Will the technological spillover network topological structure affect the diffusion of an advanced clean technology among the decision entities from the system perspective?
- If so, to what extent will the network topological structure affect the social planner’s evaluation of the effectiveness of a carbon emission policy intervention?

To answer the above research questions, we first build a systematic technology adoption model with technological spillover effect among multi-agents. Second, by assuming one agent obtaining a technological lead, we explore how the new technology is adopted by the follower agents and in the entire system upon distinct topological structures of the technological spillover network among agents. Third, through imposing carbon emission constraints on several pilot agents, we investigate how the policy intervention influences the carbon emissions of other agents without carbon emission constraints and the cumulative carbon emissions in entire system under different network topological structures.

Our work does not intend to identify the patterns or characteristics of the real-world technology adoption networks. Instead, our work abstracts away any specific technology details. With a highly simplified systematic technology adoption model and typical theoretical network topological structures, we provide a clear and intuitive understanding of the role network topological structure plays in the adoption of a new advanced technology and the evaluation of the associated environmental impacts.

The rest of this paper is organized as follows. “Research methodology and theoretical background” section introduces the methodology and theoretical background of our research, including the systematic technology adoption model, the limited foresight decision scheme, and how the new technology spills from the earlier adopters to the later adopters in the model. Section “Simulation” presents the simulation results to show how the new technology diffuses from the leader to the followers under different network structures. In “Discussion: how the network structure influences the effectiveness of a carbon emission policy intervention”, we run the simulations to investigate how the carbon emission constraints imposed on pilot agents affect the carbon emissions of other agents and thereby the entire system under different network structures. In “Conclusion and policy implications”, we conclude this paper.

## Research methodology and theoretical background

### Modeling systematic technology adoption with the ‘moving window’ decision scheme

Suppose that there are 1000 nodes in the network, each node represents an agent in the entire system. The model for each agent to optimize its own technology adoption strategies follows previous studies on modeling systematic technology adoption^8,28–32^. Suppose that in a highly simplified techno-economic system, it only provides one kind of resource (e.g. coal) and demands one kind of energy product (e.g. electricity). There are three types of energy technologies that could convert the resource into the product, namely, (a) the existing technology (T1), which is a very mature technology and has the lowest efficiency, lowest initial investment cost and the highest carbon emission; (b) the incremental technology (T2), which has the higher efficiency, higher initial investment cost and lower carbon emission than T1; (c) the revolutionary technology (T3), which is considered as the new advanced technology and featured by the highest efficiency, highest initial investment cost and zero carbon emission.

The model for each agent treats the technological change process as an endogenous process by assuming that the initial investment costs of T2 and T3 decrease as the experience of using them accumulates. This technological learning process can be described with the following Eq. (1)

1 $$\begin{array}{*{20}c} {CF_{i}^{t} = CF_{i}^{0} \times \left( {\overline{{x_{i} }}^{t - 1} } \right)^{{ - b_{i} }} , \left( {i = 2,3} \right)} \\ \end{array}$$

where, $CF_{i}^{0}$ denotes the initial unit investment cost for technology *i*. $CF_{i}^{t}$ denotes the unit investment cost for technology *i* at time *t*. $\overline{{x_{i} }}^{t}$ represents the cumulative production of technology *i* at time *t*. Here in the model, we use the cumulative production to quantify the experience (knowledge) of using one technology. $\overline{{x_{i} }}^{t}$ is calculated as the sum of the production using technology *i* at each previous time unit. $1 - 2^{{ - b_{i} }}$ denotes the learning rate of technology *i*, which indicates the cost reduction percentage when the cumulative production of technology *i* doubles. As the new advanced technology, T3 has a higher learning rate than T2. In addition, as a mature technology, T1’s investment cost remains a constant at each time *t*.

The objective function for an agent is to minimize its accumulated total cost over its one decision-making period. As shown in Eq. (2), the total cost is composed of three parts: the first part is the (discounted) total investment cost of all three technologies, where, $\delta$ represents the discount rate, $y_{i}^{t}$ represents the newly installed capacity of technology *i* at time *t*; the second part is the (discounted) total resource extraction cost, where, $C_{E}^{t}$ and $R^{t}$ denote the unit resource extraction cost and the total resource consumption at time *t*, respectively; the third part is the (discounted) total operation and maintenance cost, where, $C_{OMi}$ denotes the unit OM cost for technology *i*, $x_{i}^{t}$ denotes the production using technology *i* at time *t*.

2 $$\begin{array}{*{20}c} {{\text{min }}\mathop \sum \limits_{i = 1}^{3} \mathop \sum \limits_{t = 1}^{T} \frac{1}{{\left( {1 + \delta } \right)^{t} }}CF_{i}^{t} y_{i}^{t} + \mathop \sum \limits_{{{\text{t}} = 1}}^{{\text{T}}} \frac{1}{{\left( {1 + \delta } \right)^{t} }}C_{E}^{t} R^{t} + \mathop \sum \limits_{i = 1}^{3} \mathop \sum \limits_{t = 1}^{T} \frac{1}{{\left( {1 + \delta } \right)^{t} }}C_{OMi} x_{i}^{t} ,} \\ \end{array}$$

The objective function should subject to different constraints, as shown from Eqs. (3) to (5):

3 $$\begin{array}{*{20}c} {D^{t} \le \mathop \sum \limits_{i = 1}^{3} x_{i}^{t} , \left( {t = 1,2, \ldots ,T} \right)} \\ \end{array}$$

4 $$\begin{array}{*{20}c} {x_{i}^{t} \le C_{i}^{t} , \left( {t = 1,2, \ldots ,T} \right)\;\;\;\left( {i = 1,2,3} \right)} \\ \end{array}$$

5 $$\begin{array}{*{20}c} {x_{i}^{t} ,y_{i}^{t} \ge 0, \left( {t = 1,2, \ldots ,T} \right)\;\;\;\left( {i = 1,2,3} \right)} \\ \end{array}$$

Inequation set (3) indicates that the total production of all three technologies must satisfy its demand at each time *t*, where, $D^{t}$ denotes the annual demand at time *t*, which increases exogenously over time with a constant growth rate. Inequation set (4) indicates that the production using technology *i* cannot go beyond its total installed capacity at each time *t* (as denoted by $C_{i}^{t}$). Inequation set (5) are nonnegative constraints. For more mathematical details about the model, please refer to the Supplementary Methods.

It is noted that traditional technology adoption optimization models always assume that decision makers have perfect foresight for the future. However, in real life, decision makers need to make adaptive decisions based on the evaluation of the current market. Therefore, in the model, we assume that each agent adopts the ‘moving window’ limited foresight decision scheme^30,32,33^, which means, each agent only optimizes its technology adoption decisions within its foresight (i.e., length of the decision-making period). One decision-making period is composed of several decision-making units (normally, a decision-making unit is 10 years). With the ‘moving window’ decision scheme, taking one agent’s foresight of 50 years as an example, in the very beginning, it will make the first 50-years plan (e.g. from 2000 to 2050); however, after only one decision-making unit, it will adjust its decisions based on current market state, and make the next 50-years plan (from 2010 to 2060). There’s an overlap (of 40 years) between every two decision-making periods. In this way, the decision window (of 50 years) keeps moving forward until all the decisions for the entire decision horizon (time span of the problem) are performed.

### Topological structure of the technological spillover network

With the technological spillover effect among agents, if one agent adopts the new advanced technology earlier than the other agents (due to, e.g., R&D expenditure), its experience of using this new technology could spill over to the other agents, and makes it easier and cheaper for the other agents to adopt this new advanced and initially expensive technology. In view of this, we consider the technological spillover among agents from the network perspective. Each node represents an agent who makes technology adoption decisions with the aforementioned optimization model; each edge represents a connection between two agents, which indicates that those two agents could exchange the knowledge and experience of using the new advanced technology.

Assuming Agent *k* is connected with other *n* agents (Agent $j_{1} ,j_{2} , \cdots ,j_{n}$), due to the technological spillover among connecting agents, its total cumulative experience of using technology *i* at time *t*, $\overline{\overline{x}}_{i,k}^{t}$, now equals to Agent *k*’s own cumulative experience $\overline{x}_{i,k}^{t}$ and the experience that other *n* agents spill over to it, as described in the following equation:

6 $$\overline{\overline{x}}_{i,k}^{t} \begin{array}{*{20}c} { = \overline{x}_{i,k}^{t} + \theta \mathop \sum \limits_{m = 1}^{n} \overline{x}_{{i,j_{m} }}^{t} ,} \\ \end{array}$$

where, $\theta \in \left[ {0,1} \right]$ is a spillover rate. A higher spillover rate indicates a lower technological barrier among agents. Accordingly, the investment costs of T2 and T3 for Agent *k* are now calculated with the following Eq. (7).

7 $$\begin{array}{*{20}c} {CF_{i,k}^{t} = CF_{i,k}^{0} \times \left( {\overline{\overline{x}}_{i,k}^{t - 1} } \right)^{{ - b_{i} }} ,\;\;\; \left( {i = 2,3} \right)} \\ \end{array}$$

From the perspective of complex network, how agents are connected with each other in the entire system, i.e., the topological structure of the network among agents, shapes the channels in which new technology’s experience could spread from one agent to another. Previous researches rarely discuss this question in modeling systematic technology adoption. They commonly assume that the knowledge/experience can spillover freely among all the agents within the system, i.e., the topological structure of the network is assumed to be globally coupled^30,31^. Another strand of literature regards the technological spillover from the spatial dimension and divides the regions into core, rim and periphery areas. They point out that technology spread from the core (where a new technology is invented) to the rim, and eventually to the periphery^9,34^.

### Simulation purpose and design

With the above discussion, the novelty of this paper is that we employ typical network topological structures, namely, the regular lattices (including the globally coupled network, the nearest-neighbor coupled network, and the star coupled network), the random network (considering different connecting probability), and the scale-free network, to explore how the technological spillover network structure affects the systematic technology adoption and carbon emissions.

As mentioned in the introduction, we aim to address two research questions. To this end, we conduct the study with numerical simulations in proceeding sections. To answer the first question, we will run simulations to investigate how the new advanced technology diffuses from the leader to the followers and how it is adopted in the entire system under different technological spillover network structures. To answer the second question, we will run simulations to investigate how the carbon emission constraints imposed on pilot agents influence the carbon emissions of the other agents and the entire system when the topological structure of the technological spillover network varies.

## Simulation

In this section, we assume that there are 1000 nodes in the network (i.e., 1000 agents in the entire system). One of the agents (let it be Agent 1) holds the technological lead in the new advanced technology, which means, it has already possessed some knowledge and experience of T3. Naturally, Agent 1 will adopt T3 earlier than the other agents and act as the leader in the network. Other 999 agents are thus the followers in the network. Each agent makes technology adoption decisions with the optimization model introduced in the previous section, and each agent also adopts the ‘moving window’ limited foresight decision scheme with the same foresight of 50 years, which follows previous literature^33^. The decision horizon is 100 years. Other initial parameters for all the agents are the same (as shown in Table S1 in the Supplementary Methods), except that for Agent 1, $\overline{x}_{3}^{0} = 50$. The settings of the parameters’ initial values also follow previous researches^29^. The simulation settings are deliberately simplified so that we can observe more transparently how the new advanced technology diffuses from the leader to the followers in the network with different topological structures. It should be noted that, in our model, each technology essentially represents a cluster of related technologies with the infrastructure being in the center. The diffusion of a cluster of new technology can span up to a century . It takes even a longer time to evaluate the climate impact of a new technology. Therefore, we set the simulation time to 100 years.

**Figure 1:**
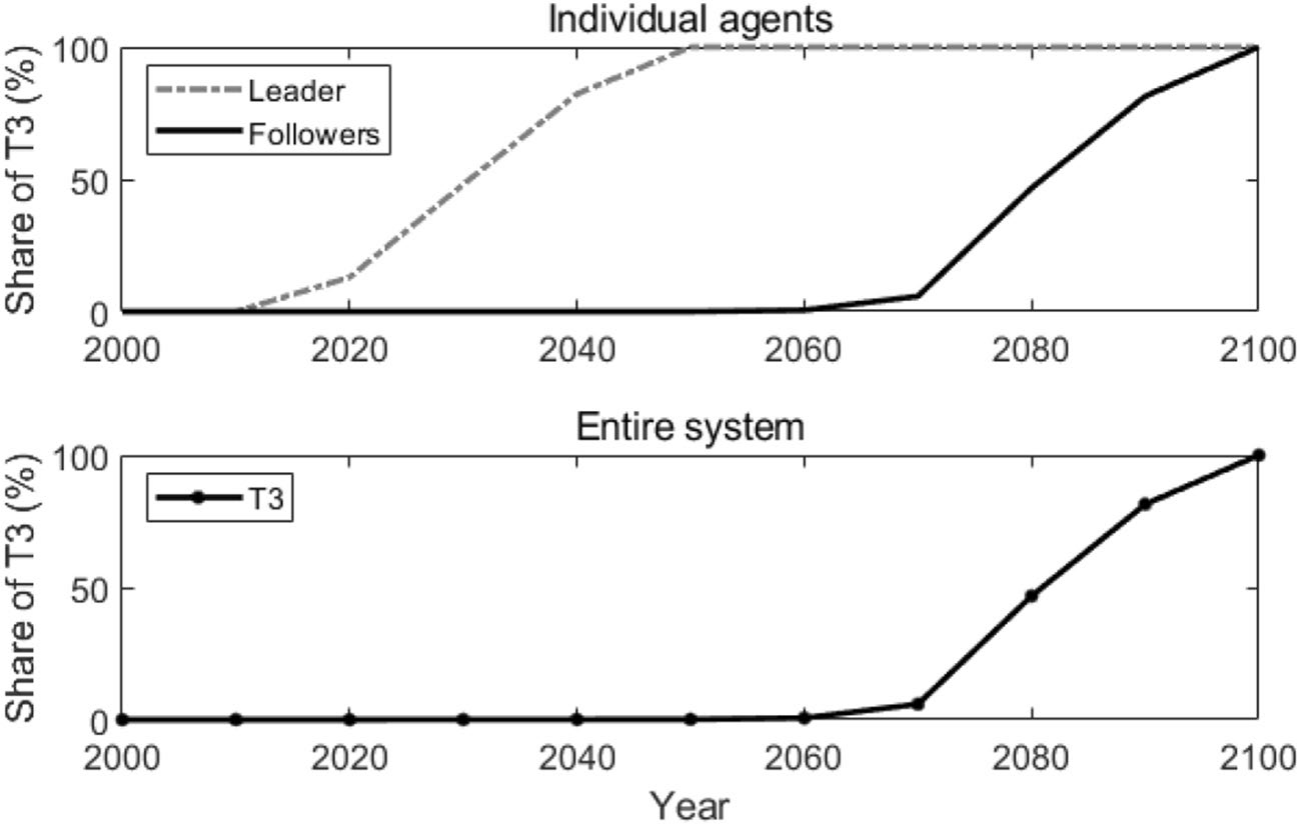
Reference scenario in “Simulation”. Lines in the figure represent how the shares of T3 change over time for the leader and the followers individually (top panel), and in the entire system (bottom panel).

Figure 1 shows how T3 is adopted by the leader (Agent 1) and the followers (other agents) individually in the reference scenario when all the agents make decisions independently without exchanging any information or knowledge with each other, i.e., there’s no technological spillover among agents. Since the leader holds the technological lead in the new advanced technology T3, it adopts T3 around the year 2020, while the followers adopt T3 around the year 2080 (when the share of T3 reaches 10%). How the new advanced technology T3 is adopted in the entire system is also shown in Fig. 1. Due to the fact that the followers take up the overwhelming majority number of all the agents, T3’s adoption in the entire system seems the same as the followers, which is around the year 2080.

The no-spillover scenario can also be viewed as the investment failure scenario, since the followers cannot acquire the knowledge of the new technology from the leader and continue to use the old technology until the new technology becomes cost-effective for them. In the following simulations, we assume that the technological spillover effect exists among the agents. We will investigate how T3 diffuses from the leader to the followers when the technological spillover network is a regular lattice, a random network, or a scale-free network, respectively.

### Regular lattices

#### Globally coupled network

**Figure 2:**
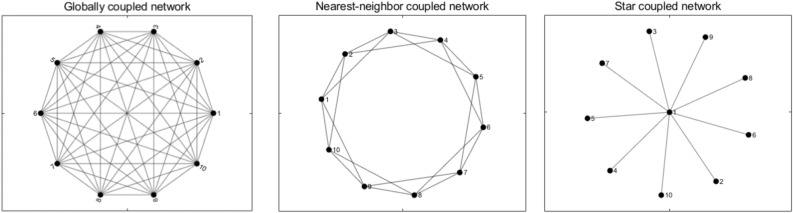
Illustration of three typical regular lattices.

In a globally coupled network, all nodes are connected with each other, as illustrated in the left panel of Fig. 2, taking a network with 10 nodes as an example.

**Figure 3:**
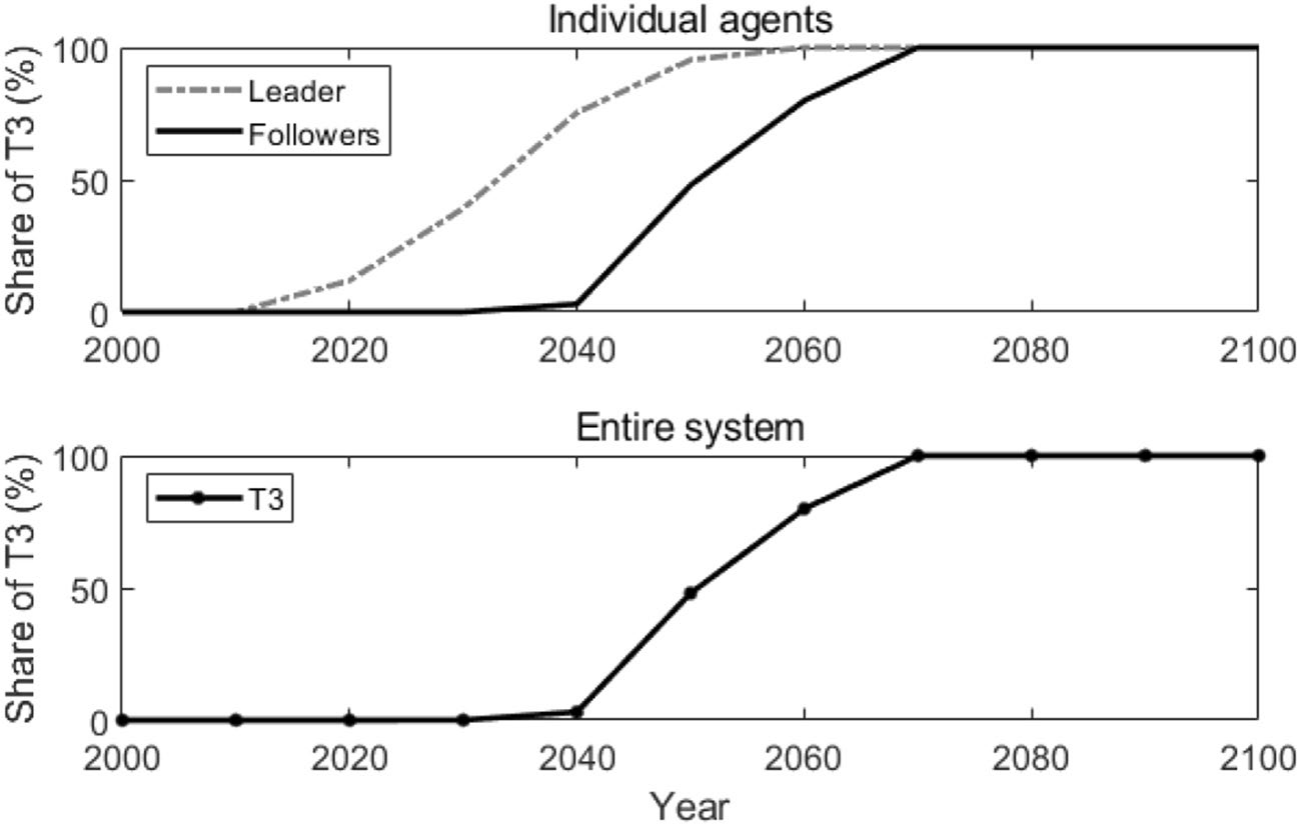
Adoption of T3 in the globally coupled network. Lines in the figure represent how the shares of T3 change over time.

Since all the 1000 agents in the network are connected with each other, every follower agent acquires the same information at each time *t*, and thus makes the same technology adoption decisions. Figure 3 shows how T3 is adopted by the leader and the followers individually and in the entire system. Compared with the results in the reference scenario, T3 is adopted 30 years earlier by the followers and in the entire system in the globally coupled network.

**Figure 4:**
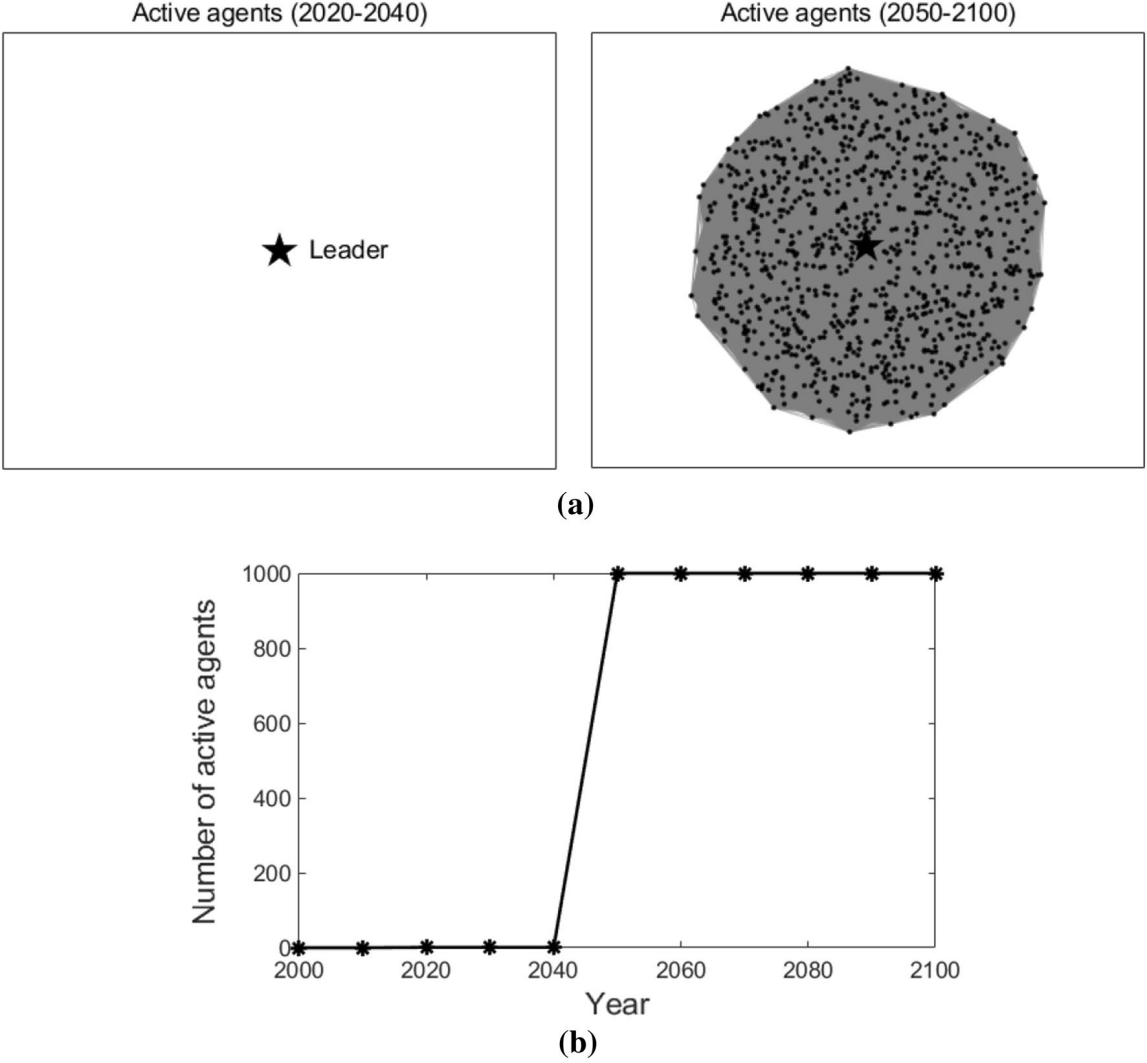
Active agents and the number of active agents in the globally coupled network. Sub-figure (**a**) presents active agents at different times, and sub-figure (**b**) plots the number of active agents at different times. In sub-figure (**a**), the star-shaped node represents the leader, the black dot nodes represent the followers, and the grey lines represent the edges that connect the agents.

Figure 4 illustrates the agents in the network who adopt T3 at different times (as shown in Fig. 4a), agents who do not adopt T3 are removed from the figures) and the number of agents that adopt T3 at different times (as shown in Fig. 4b)). To make the description clearer, agents who adopt T3 at time *t* are named active agents at time *t*. The leader is highlighted with a star-shaped node, while the followers are represented with dot nodes. The edges among the nodes are shown with grey lines in the figures, which represent the connections among agents. As we can observe in Fig. 4, in the early stage (2000–2010), no agent adopts T3; from the year 2020 to 2040, only the leader adopts T3; after the year 2050, all the followers start to adopt T3. This is because, in a globally coupled network, all the followers are connected to the leader and with each other. Therefore, they adopt the new advanced technology at the same time.

#### Nearest-neighbor coupled network

In a nearest-neighbor coupled network, each node only connects with its K neighbor nodes (K/2 neighbor nodes on each side), as illustrated in the middle panel of Fig. 2, taking 10 nodes and K = 4 as an example.

**Figure 5:**
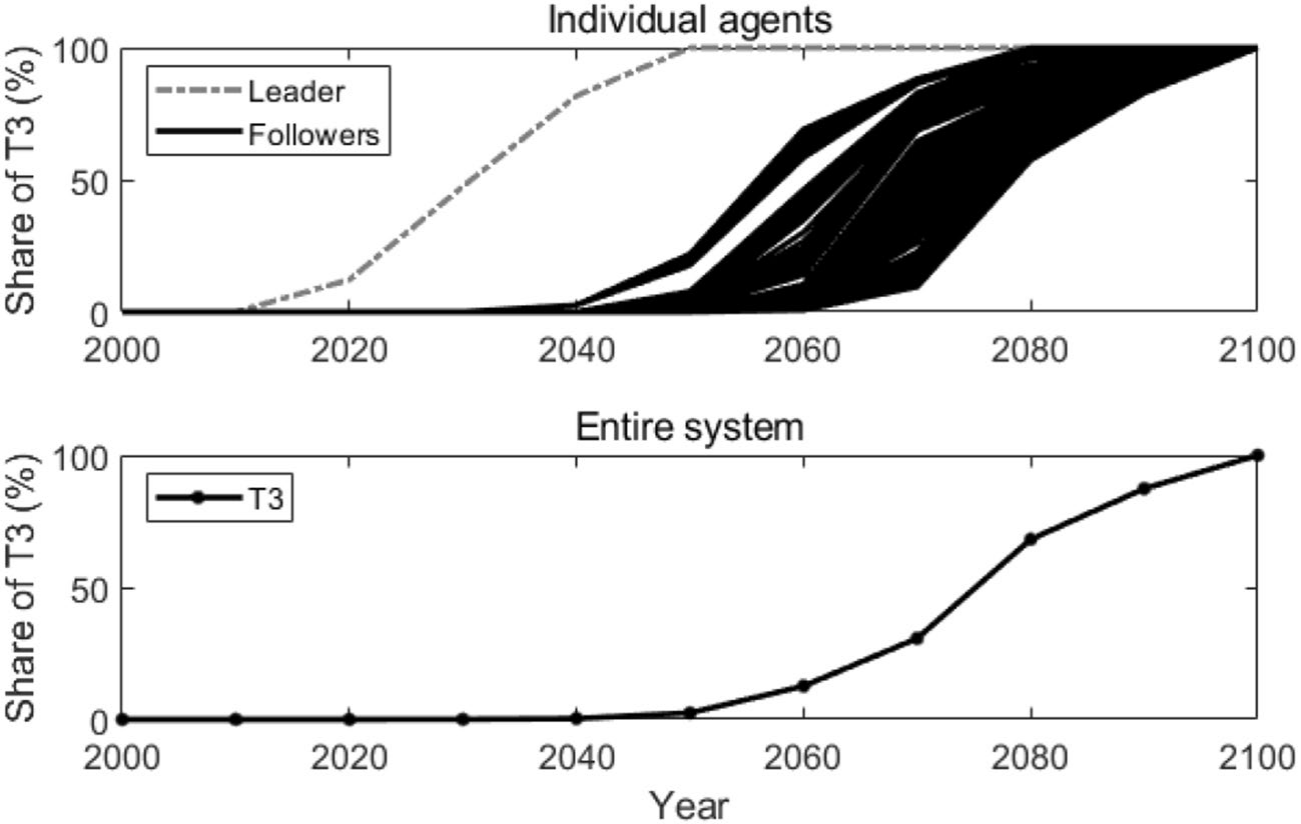
Adoption of T3 in the nearest-neighbor coupled network. Lines in the figure represent how the shares of T3 change over time.

In our simulation, we let K = 100, which means each agent connects with its 100 neighbor agents (50 neighbor agents on each side). Figure 5 shows how T3 is adopted by each individual agent and in the entire system. The followers adopt T3 gradually since they acquire the knowledge of the new advanced technology at different times. The earliest and the latest adoption of T3 for the followers are around the years 2050 and 2080. There is an adoption time lag of 30 years among the followers. In the entire system, T3 is adopted around the year 2060, 20 years earlier compared with the results in the reference scenario.

**Figure 6:**
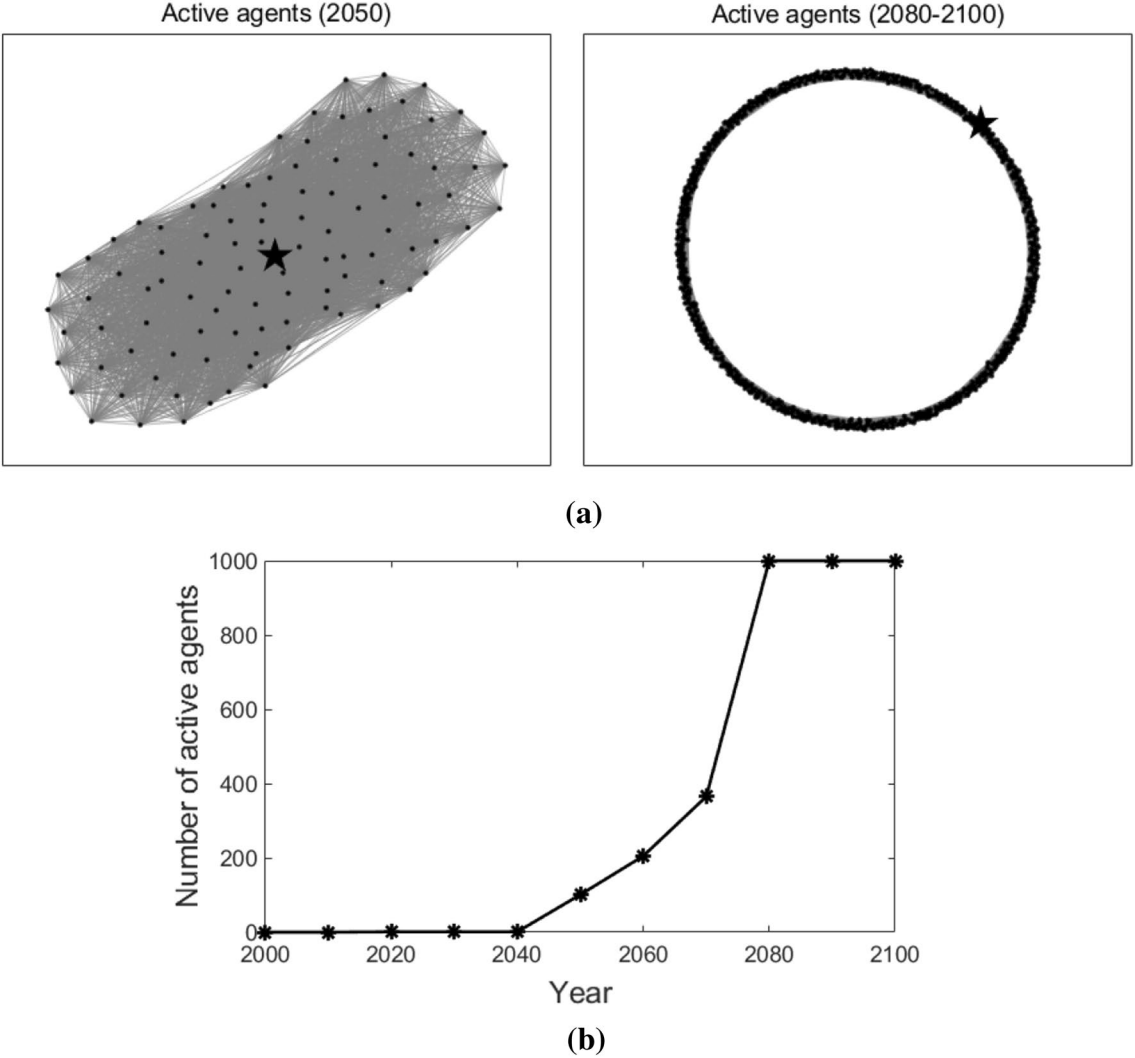
Active agents and the number of active agents in the nearest-neighbor coupled network. Sub-figure (**a**) presents active agents at different times, and sub-figure (**b**) plots the number of active agents at different times. In sub-figure (**a**), the star-shaped node represents the leader, the black dot nodes represent the followers, and the grey lines represent the edges that connect the agents.

Figure 6 illustrates the active agents at different times (as shown in Fig. 6a) and the number of active agents that changes over time (as shown in Fig. 6b) in the nearest-neighbor coupled network. The star-shaped node also represents the leader, and the dot nodes also represent the followers. As we can observe, in 2050, not many followers adopt T3. After that, more and more followers start to adopt T3 and the number of active agents keeps increasing. After the year 2080, T3 is adopted by all the agents. This is because, in a nearest-neighbor coupled network, each agent connects with its neighbor nodes. Followers can only acquire the knowledge of T3 gradually from its neighbors. As a result, the number of active agents also increases gradually.

#### Star coupled network

In a star coupled network, there is one node that occupies the center of the star and all the other nodes only connect with the center node, as illustrated in the right panel of Fig. 2, taking 10 nodes as an example.

**Figure 7:**
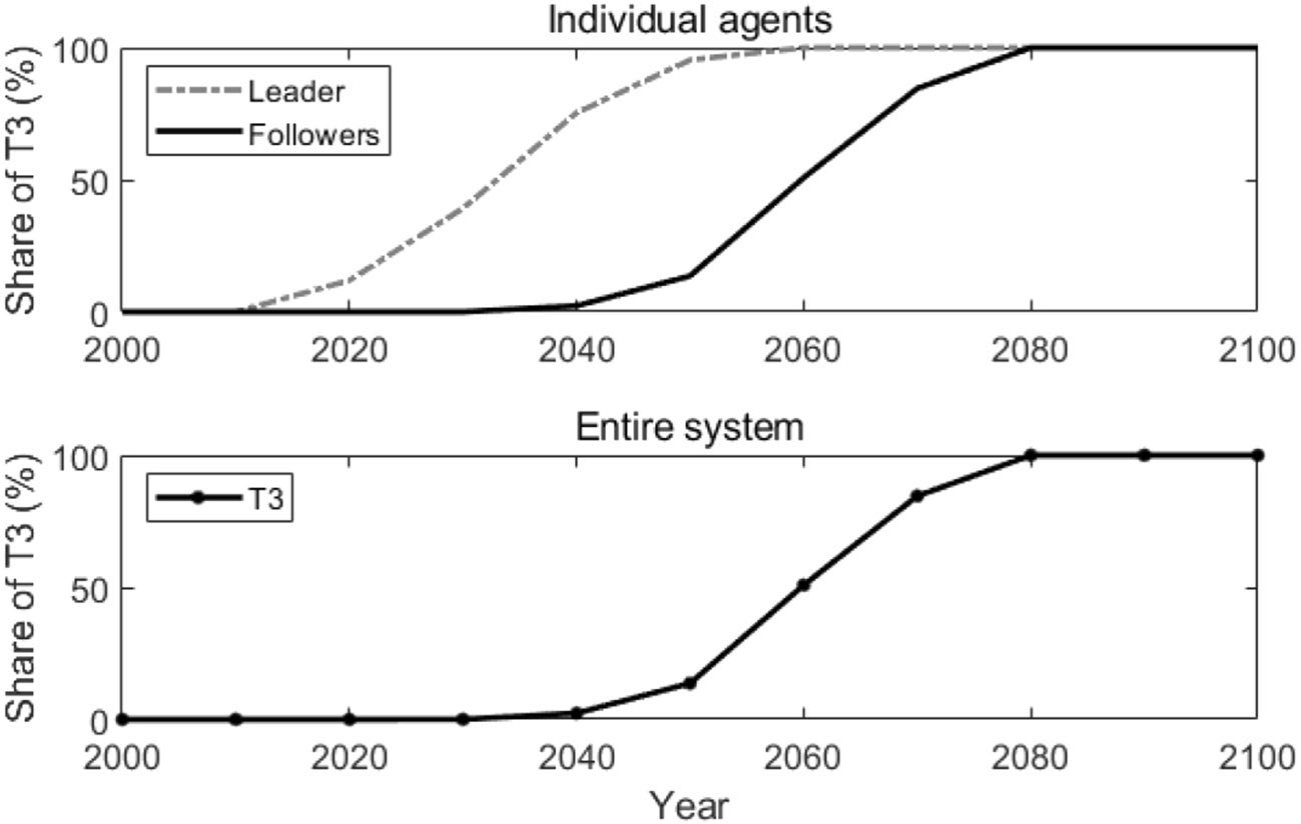
Adoption of T3 in the star coupled network. Lines in the figure represent how the shares of T3 change over time.

In our simulation, Agent 1 has the technological lead, therefore, it is assumed intuitively to be the center node, and all the followers connect with and only with it. As a matter of fact, since all the followers are identical and only connect with the leader, they also acquire the same knowledge at each time *t*, and thereby make the same technology adoption decisions as well. Figure 7 shows how T3 is adopted by the leader and the followers individually and in the entire system. Compared with the results in the reference scenario, T3 is adopted 30 years earlier by the followers and in the entire system.

**Figure 8:**
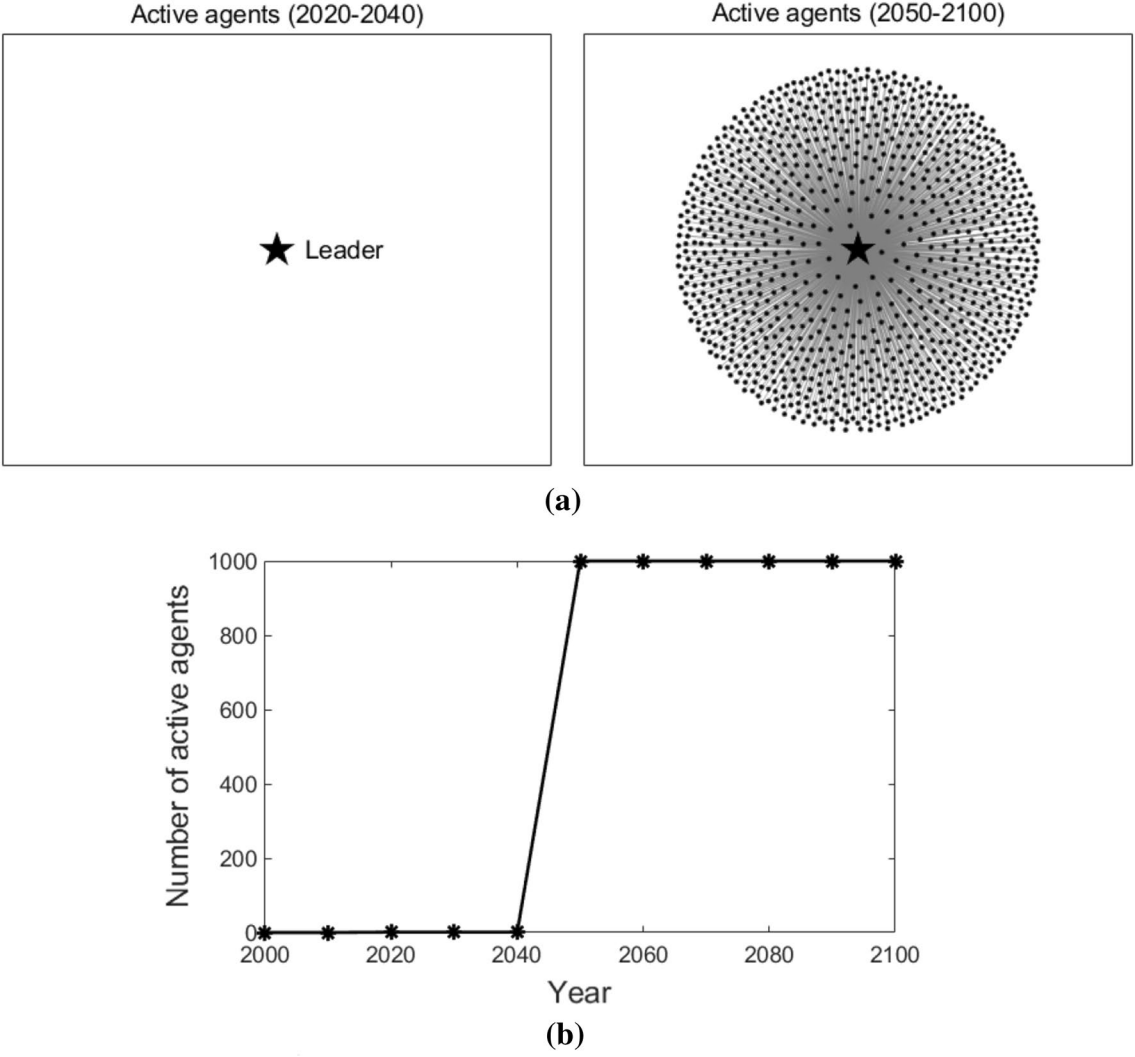
Active agents and the number of active agents in the star coupled network. Sub-figure (**a**) presents active agents at different times, and sub-figure (**b**) plots the number of active agents at different times. In sub-figure (**a**), the star-shaped node represents the leader, the black dot nodes represent the followers, and the grey lines represent the edges that connect the agents.

Figure 8 illustrates the active agents at different times and the number of active agents that changes over time in the star coupled network. From the year 2020 to 2040, only the leader adopts T3. After the year 2050, all the followers start to adopt T3. This is because, in a star coupled network, the followers only connect to the leader and can acquire the knowledge of T3 directly from the leader. Therefore, similar to the globally coupled scenario, they adopt T3 quickly and at the same time.

### Random networks

In this subsection, we will run simulations under the assumption that the technological spillover network among all the 1000 agents is a random network. We follow the study conducted by Erdős and Rényi to construct the *ER* random graph $G_{N,p}^{ER}$, where each pair of nodes is assumed to be connected with a probability *p*^35^. To capture its dynamics, we also assume that the static random networks among all the nodes are different at different times.

According to the random graph theory, suppose that there are *N* nodes in a network, there is a critical probability $p_{c} = 1/N$. Also, when $p \ge {\text{ln}}\left( N \right)/N$, almost any graph in the ensemble $G_{N,p}^{ER}$ is totally connected^35,36^. Therefore, in our research, we will run different simulations with the connecting probability *p* equals to 0.001, 0.01, and 0.1, respectively. We will explore how the new advanced technology diffuses in the randomly connected networks in the following three scenarios: (a) when the connecting probability is comparatively low; (b) when there exists a giant component in the network; and (c) when the random network is always connected.

**Figure 9:**
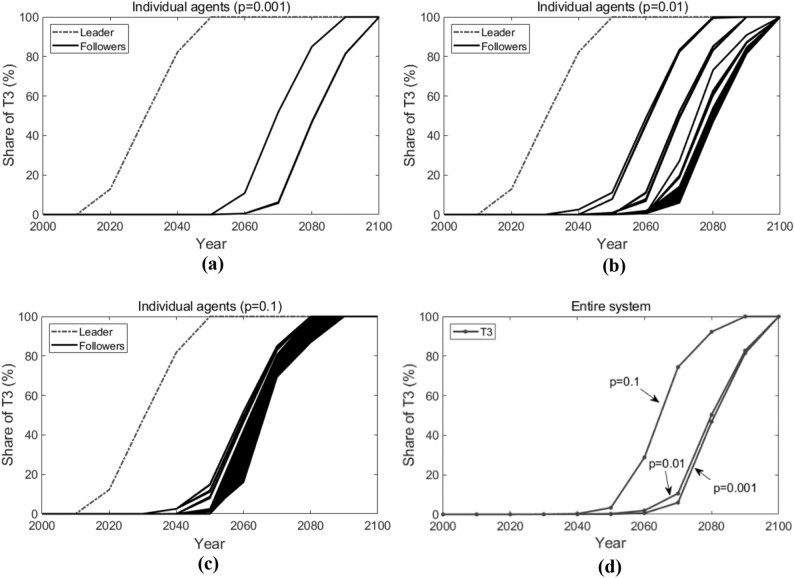
Adoption of T3 with different connecting probabilities. Lines in the figure represent how the shares of T3 change over time.

Figure 9 shows how T3 is adopted by each individual agent (sub-figures (a), (b) and (c), in which the dashed line represents the leader and the solid lines represent the followers) and in the entire system (sub-figure (d)) when the connecting probability $p$ varies. When $p = 0.001$, the followers adopt T3 around the years 2060 to 2080. When $p = 0.1$, the followers generally adopt T3 earlier, around the years 2050 to 2060. In the entire system, when *p* increases from the 0.001 to 0.1, the adoption of T3 is advanced from the year 2080 to the year 2060.

**Figure 10:**
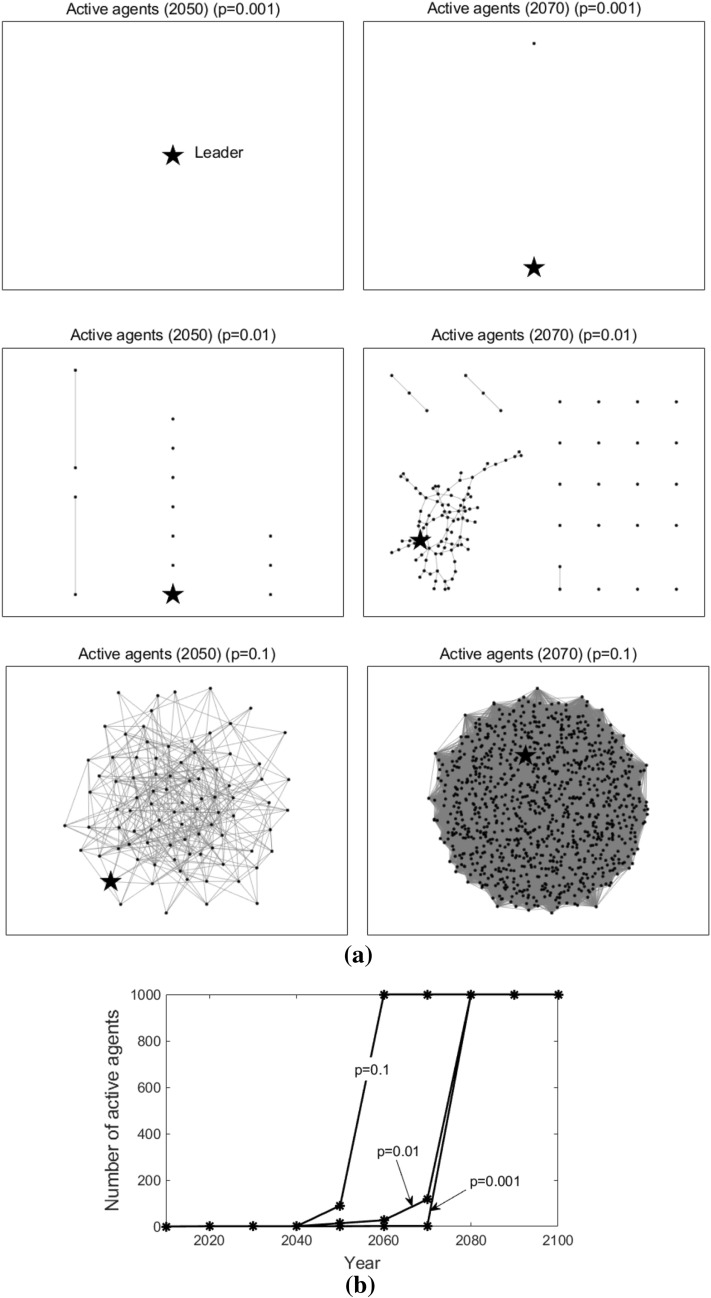
Active agents and the number of active agents in the random networks. Sub-figure (**a**) presents active agents at different times, and sub-figure  (**b**) plots the number of active agents at different times. In sub-figure (**a**), the star-shaped node represents the leader, the black dot nodes represent the followers, and the grey lines represent the edges that connect the agents.

Figure 10 shows the active agents at different times (here we present the active agents in 2050 and 2070 as illustrations) and how the number of active agents changes over time when $p$ varies. Likewise, the star-shaped node represents the leader, the black dot nodes represent the followers, and the grey lines represent the edges that connect the agents. As we can observe, when $p = 0.001$, in 2050, only the leader adopts T3; in 2070, only one follower starts to adopt T3. When $p = 0.01$, in 2050, several followers adopt T3; in 2070, more followers adopt T3. When $p = 0.1$, in 2050, about 90 followers start to adopt T3; in 2070, all followers adopt T3. Note that, in the figure, there are independent dot nodes (without the connection to any other nodes). This is because, in our simulation, the random network structure varies at each time *t*. Previous connected nodes may not be connected currently, but they have already acquired the knowledge of T3 through previous connections. From Fig. 10b), we can conclude that, the higher the connecting probability is, the faster the number of active agents increases, that is, the earlier the followers adopt the new advanced technology T3.

In a word, as the connecting probability $p$ increases, more and more followers tend to initiate their adoption of the new advanced technology earlier. It is in accordance with our intuition that with a larger connecting probability, more follower agents could be topologically connected with the leader agent, and they can benefit from the technological spillover effect more straightforwardly.

### BA scale-free network

In this subsection, we will run simulations under the assumption that the technological spillover network among all 1000 agents is a BA scale-free network. The ER random graph neglects two most important features of the network in the real world: growth and preferential attachment^37^.

**Figure 11:**
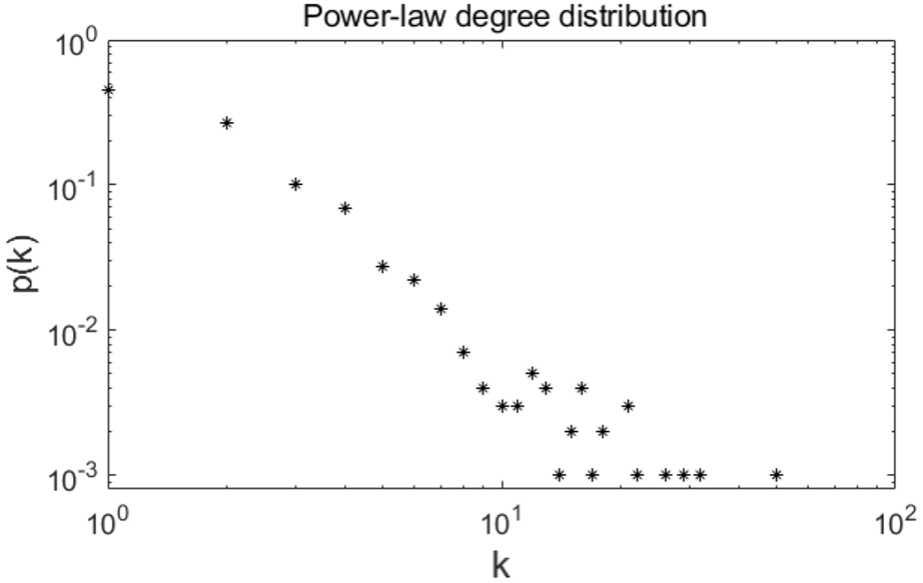
The power-law degree distribution of the constructed scale-free network.

Following the approach proposed by Albert and Barabási, we construct the scale-free network with the following rules^38^:

- Initially, there exists a connected network with $m_{0}$ nodes. We assume that there are $m_{0} = 10$ nodes connecting with each other randomly in the initial network, including the leader Agent 1. The remaining 990 nodes are pending to be added to the network. In each iteration, we add a new node with *m* edges that link the new node to *m* present nodes in the system. *m* should be less than $m_{0}$, and we let *m* = 5.
- When choosing the nodes to which the new node connects, the probability of the new node connecting with a present node *i* in each iteration is computed with the following equation:
  8 $$\begin{array}{*{20}c} {p_{i} = \frac{{k_{i} }}{{\mathop \sum \nolimits_{j} k_{j} }},} \\ \end{array}$$

where, $k_{i}$ is the degree of present node *i*, $\mathop \sum \limits_{j} k_{j}$ represents the sum of the degrees of all present nodes. Figure 11 shows the power-law degree distribution of the BA scale-free network we constructed.

**Figure 12:**
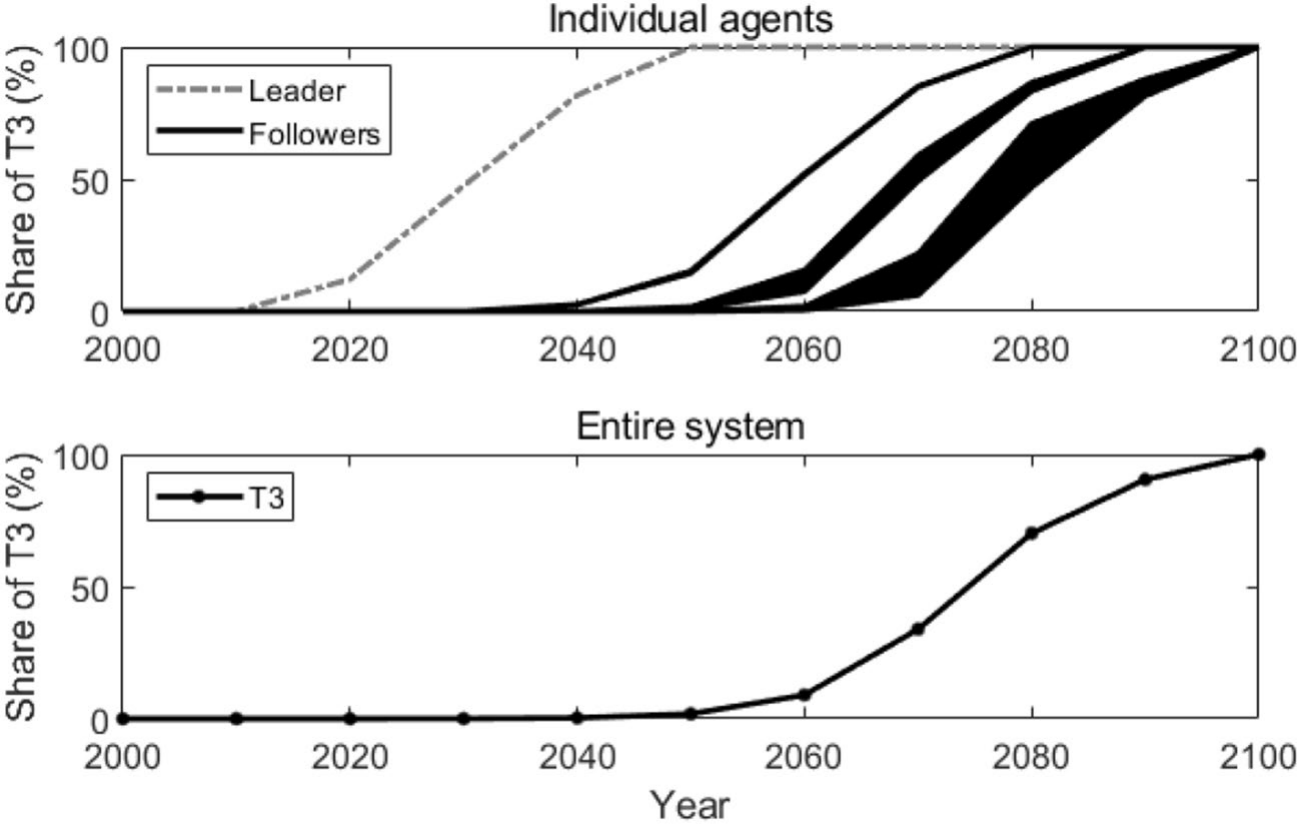
Adoption of T3 in the scale-free network. Lines in the figure represent how the shares of T3 change over time.

Figure 12 presents how T3 is adopted by individual agents and in the entire system. As we can observe, the followers’ adoption time of T3 ranges from the year 2050 to the year 2080. In the entire system, T3 is adopted around the year 2070, only 10 years earlier compared with what suggested in the reference scenario.

**Figure 13:**
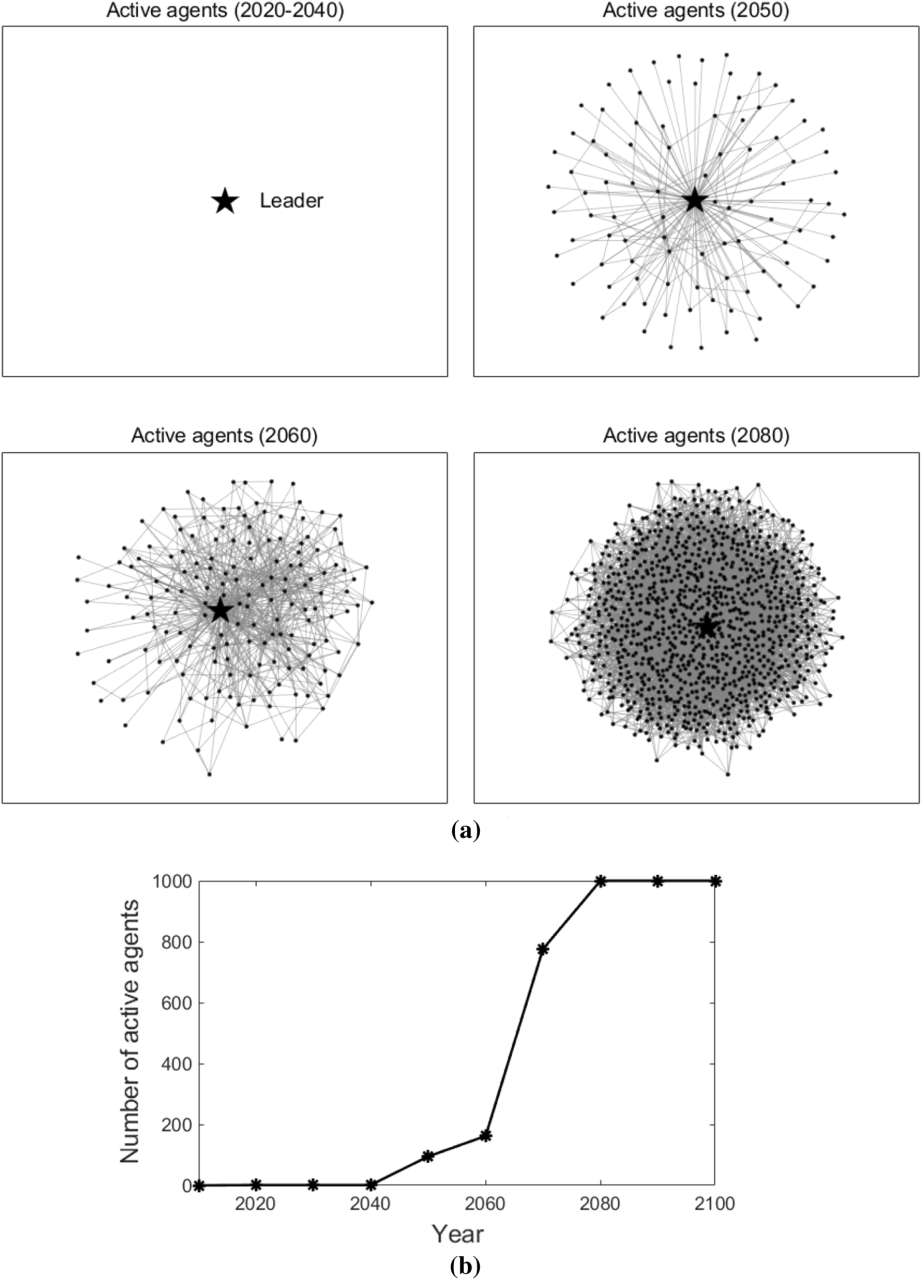
Active agents and the number of active agents in the scale-free network. Sub-figure (**a**) presents active agents at different times, and sub-figure (**b**) plots the number of active agents at different times. In sub-figure (**a**), the star-shaped node represents the leader, the black dot nodes represent the followers, and the grey lines represent the edges that connect the agents.

Figure 13 illustrates the active agents at different times and how the number of active agents changes over time in a scale-free network. As we can see, from the year 2020 to 2040, only the leader adopts T3; from the year 2050 to 2070, more and more followers start to adopt T3; and after the year 2080, all the followers adopt T3. This is because, in the scale-free network, most edges are connected to some nodes, i.e., a large number of agents are connected to only some agents, while the other agents are only connected to few agents. Those followers that link directly with the leader will adopt T3 earlier than the followers that are more distant from the leader.

With the discussion above, we can draw the conclusion that, the topological structure of the technological spillover network among agents plays a crucial part in the adoption and diffusion of the new advanced technology among agents and in the entire system. Some network topological structures favor the adoption and diffusion of the new technology (e.g., the globally coupled network), while others may cause adoption time lags among the follower agents (e.g., the random network and the scale-free network), and therefore delay the adoption of the new technology in the entire system. The implication of our simulation is that, in the real world, the network topological structure among decision entities might be complex. It is crucial for the social planner to take into consideration of the network structure when evaluating the diffusion process of an innovation.

In the next section, we will discuss the implication of our research further. We will examine whether different network topological structures influence the effectiveness of a carbon emission constraint policy.

## Discussion: how the network structure influences the effectiveness of a carbon emission policy intervention

In this section, we will investigate how the carbon emission constraints imposed on given nodes (pilot agents) affect the carbon emissions of other agents and thereby the entire system under different topological structures of the technological spillover network.

The carbon emission constraint policy requires as follows, among the 1000 agents in the network, 10 of them are chosen as pilot agents and imposed with carbon emission constraints (those 10 agents are chosen randomly). It doesn’t matter what specific type of carbon emission constraints they are facing (it could be carbon taxes, or carbon trades, etc.), the carbon emission constraints only require that for every pilot agent, by the target year 2050, its yearly carbon emission must decline to a lower level than that in its base year 2000, on the premise that annual production must meet the growing annual demand. The carbon emission coefficients for the unit production of T1 and T2 are 0.8 and 0.64, respectively, which also follows previous works^8^. As for the new advanced technology T3, it is assumed to be a zero-emission clean technology. Other settings for all the agents are exactly the same and also as shown in Table S1 in the Supplementary Methods. Each agent also makes technology adoption decisions with the optimization model introduced in “Research methodology and theoretical background”. In addition, the nearest-neighbor coupled network and the scale-free network are constructed according to the same rules as introduced in “Simulation”; for the star coupled network, we also assume a pilot agent occupies the center of the star; the random network is constructed with the connecting probability $p = 0.01$.

**Figure 14:**
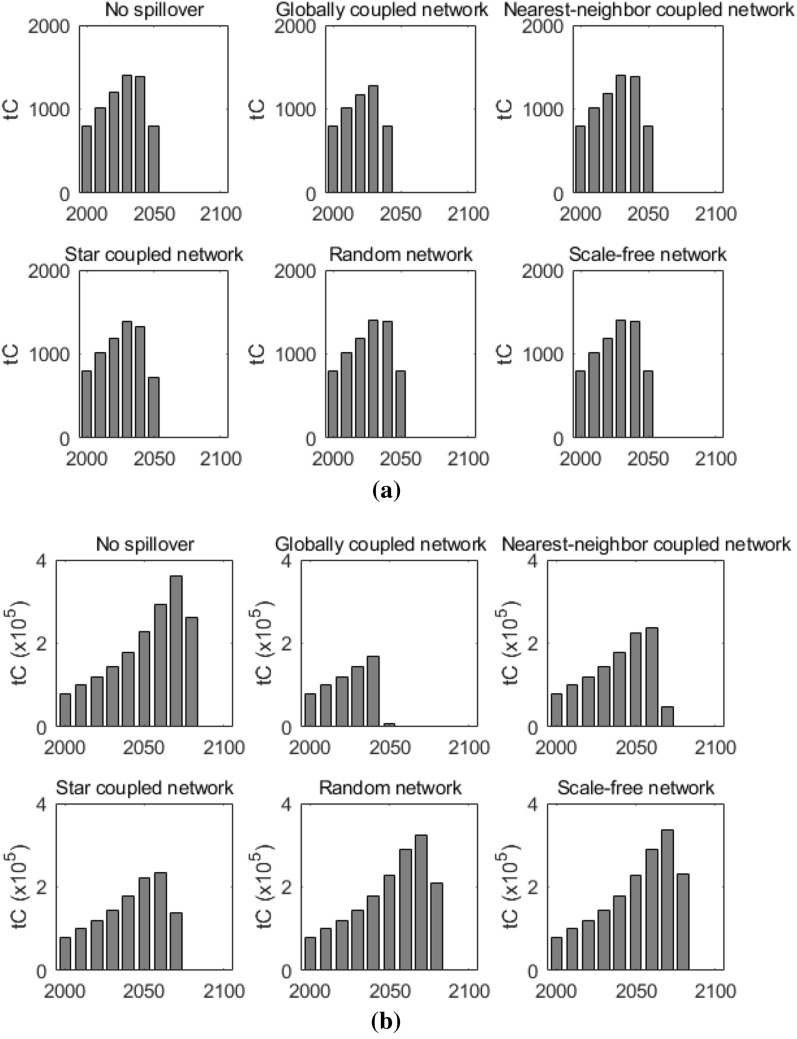
Total yearly carbon emissions for pilot agents and other agents. Sub-figure (**a**) presents total yearly carbon emissions for pilot agents, and sub-figure (**b**) presents total yearly carbon emissions for other agents. The horizontal axis represents the year, and the vertical axis represents the carbon emission. In sub-figure (**a**), the carbon emissions for all pilot agents are aggregated yearly. In sub-figure (**b**), the carbon emissions for all the other agents are also aggregated yearly.

Figure 14 shows the total yearly carbon emissions for all pilot agents (agents with emission constraints) and the total yearly carbon emissions for all the other agents (agents without emission constraints) under different network topological structures. The results when there’s no technological spillover among agents are also shown in the figures for comparison. As we can observe, if there’s no technological spillover among agents, the total yearly carbon emissions of other agents peak around the year 2070 and reach at a comparatively higher level. When there is technological spillover among agent, under different network topological structures, the total yearly carbon emissions of other agents generally decrease, but to different extents. In the target year 2050, the lowest total yearly carbon emission for other agents occurs in the globally coupled network. In the random network and the scale-free network, the total yearly carbon emissions for other agents are about 1.36 times that in the globally coupled network. As for the pilot agents, different network topological structures have little influence on their total yearly carbon emissions. In the target year 2050, as required by the policy intervention, the total yearly carbon emissions for all pilot agents under different network topological structures decrease to lower levels than those in their base year 2000. Specifically, in the globally coupled network, the total yearly carbon emission for all pilot agents even decreases to zero in the target year. The results are due to the following reasons: for the pilot agents, since they are restricted by the carbon emissions constraints, they have to accelerate their transition to the new clean technology to lower their emissions. For the other agents, with the technological spillover effect, they can generally acquire the knowledge of T3 earlier and adopt T3 earlier, however, to different extents, due to various connecting formats under various network structures. This suggests that, carbon emission constraints on pilot agents can not only reduce the carbon emissions of pilot agents, due to the technological spillover effect, they also have impacts on other agents in the system, even though they do not face carbon emission constraints. However, the degrees of the impacts are different under various network topological structures.

**Figure 15:**
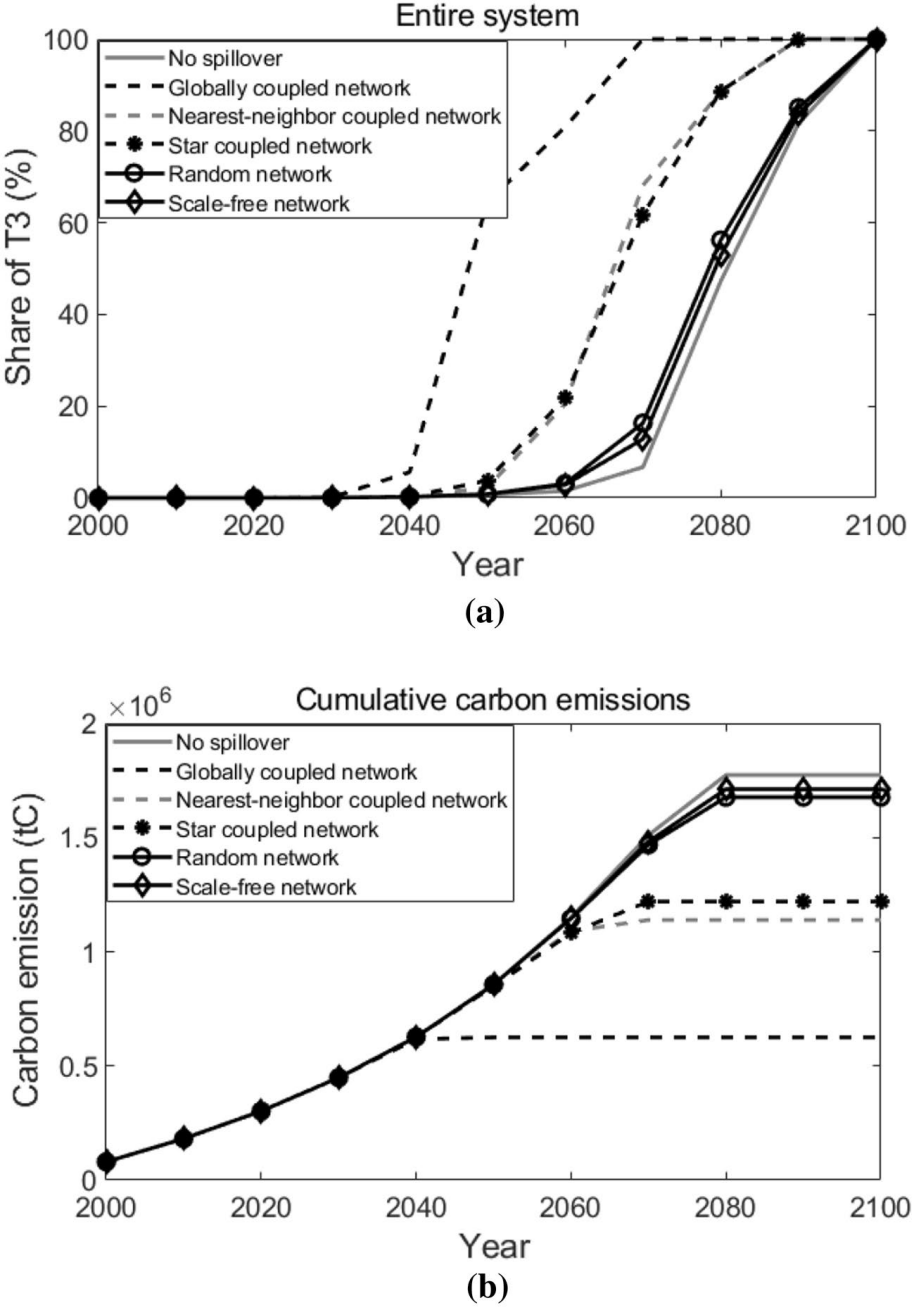
Adoptions of T3 and the cumulative carbon emissions in the entire system after policy intervention. In sub-figure (**a**), lines represent the diffusion of T3. (**a**) Adoptions of T3, (**b**) Cumulative carbon emissions.

Figure 15 shows the adoptions of T3 and the cumulative carbon emissions in the entire system under different network topological structures. Generally speaking, the years T3 appear in the entire system vary largely under different network topological structures, and so do the cumulative carbon emissions over the 100 years decision horizon. In the globally coupled network, T3 is adopted in the entire system around the year 2050, whereas in the random network or in the scale-free network, T3 is adopted in the entire system around the year 2070, very similar to the situation when there’s no spillover among agents. Consequently, the cumulative carbon emissions over the decision horizon in the random network and the scale-free network are about 2.68 and 2.74 times the value in the globally coupled network. The spread between the highest cumulative carbon emission (in the scale-free network) and the lowest cumulative carbon emission (in the globally coupled network) is $1.09 \times 10^{6}$ tC.

Next, we will do sensitivity analyses on the following factors to examine how these important factors in our simulation influence the results in our research.

**Figure 16:**
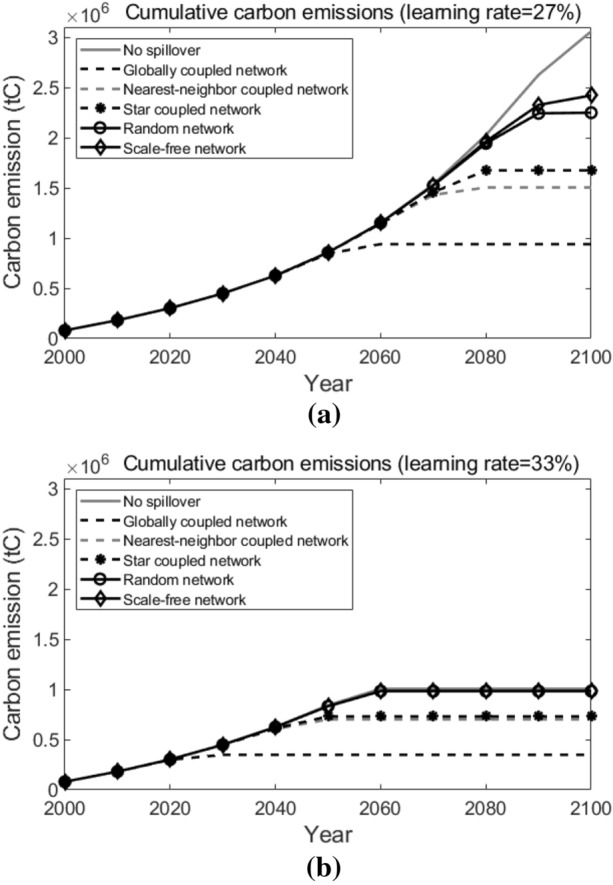
Sensitivity analysis on the learning rate. Sub-figure (**a**) presents the results when learning rate = 27%, and sub-figure (**b**) presents the results when learning rate = 33%.

**Figure 17:**
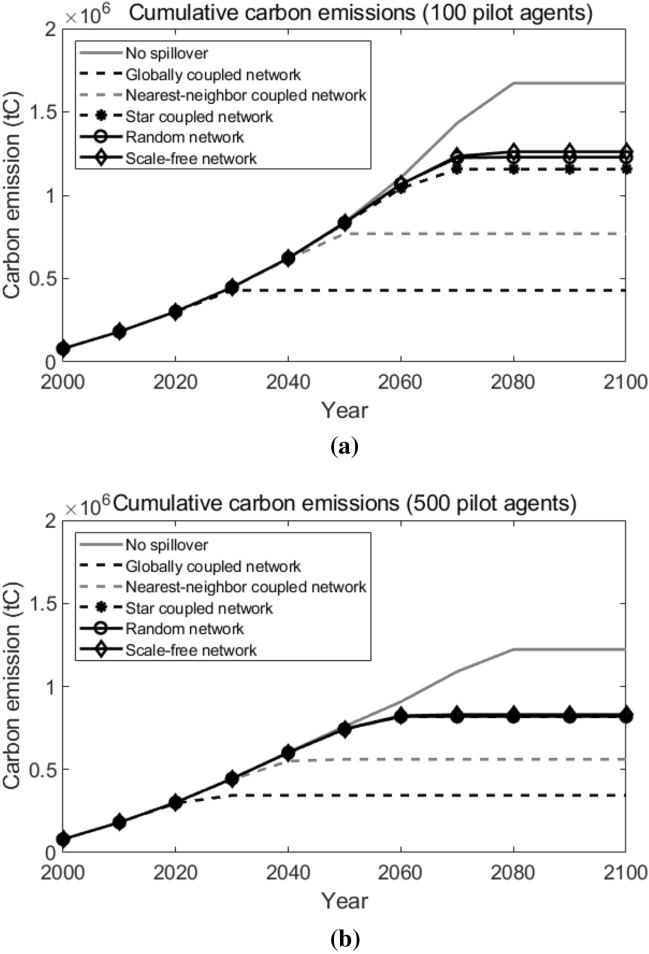
Sensitivity analysis on the number of pilot agents. Sub-figure (**a**) presents the results when the number of pilot agents = 100, and sub-figure (**b**) presents the results when the number of pilot agents = 500.

**Figure 18:**
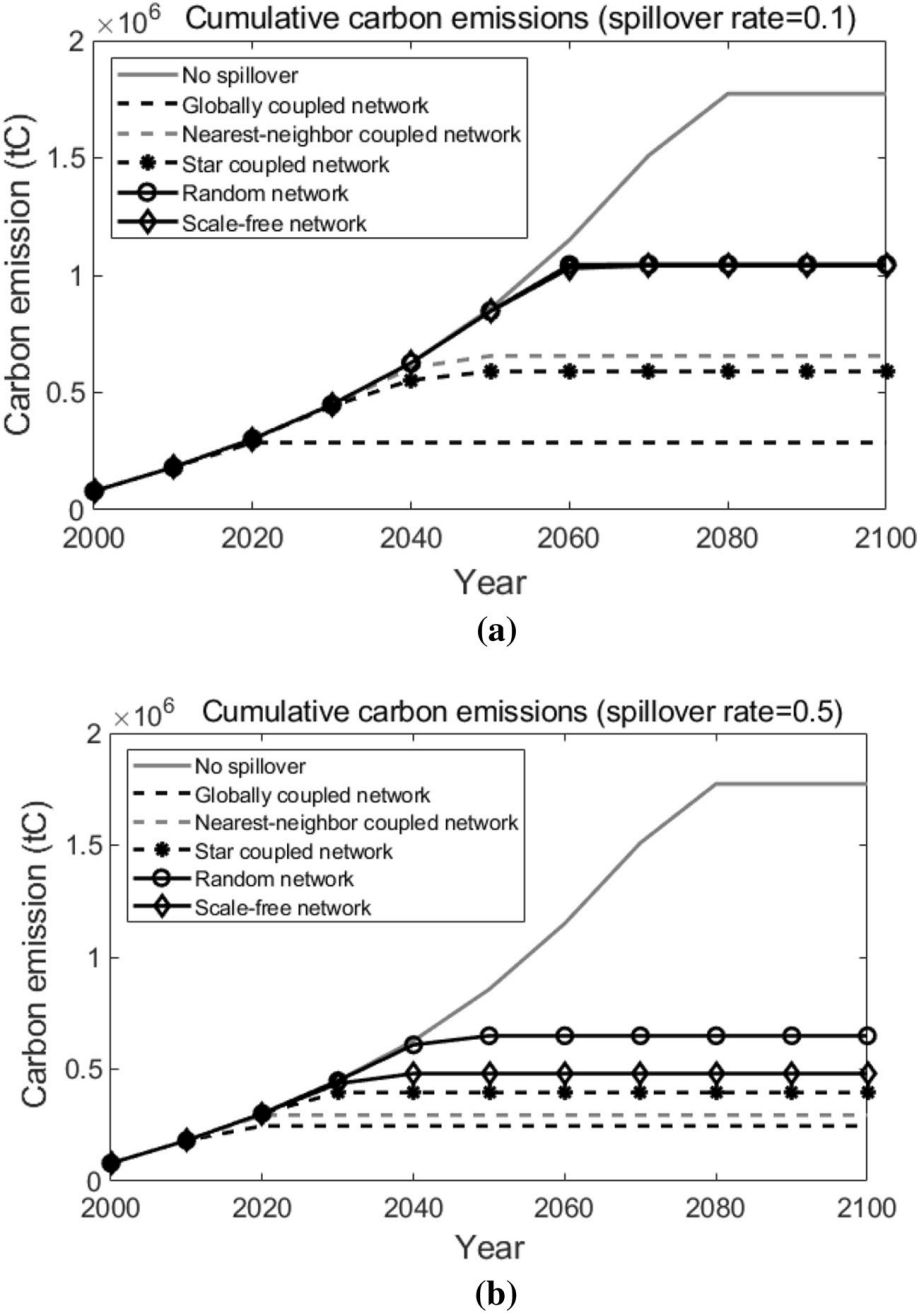
Sensitivity analysis on the spillover rate. Sub-figure (**a**) presents the results when the spillover rate = 0.1, and sub-figure (**b**) presents the results when the spillover rate = 0.5.

**Figure 19:**
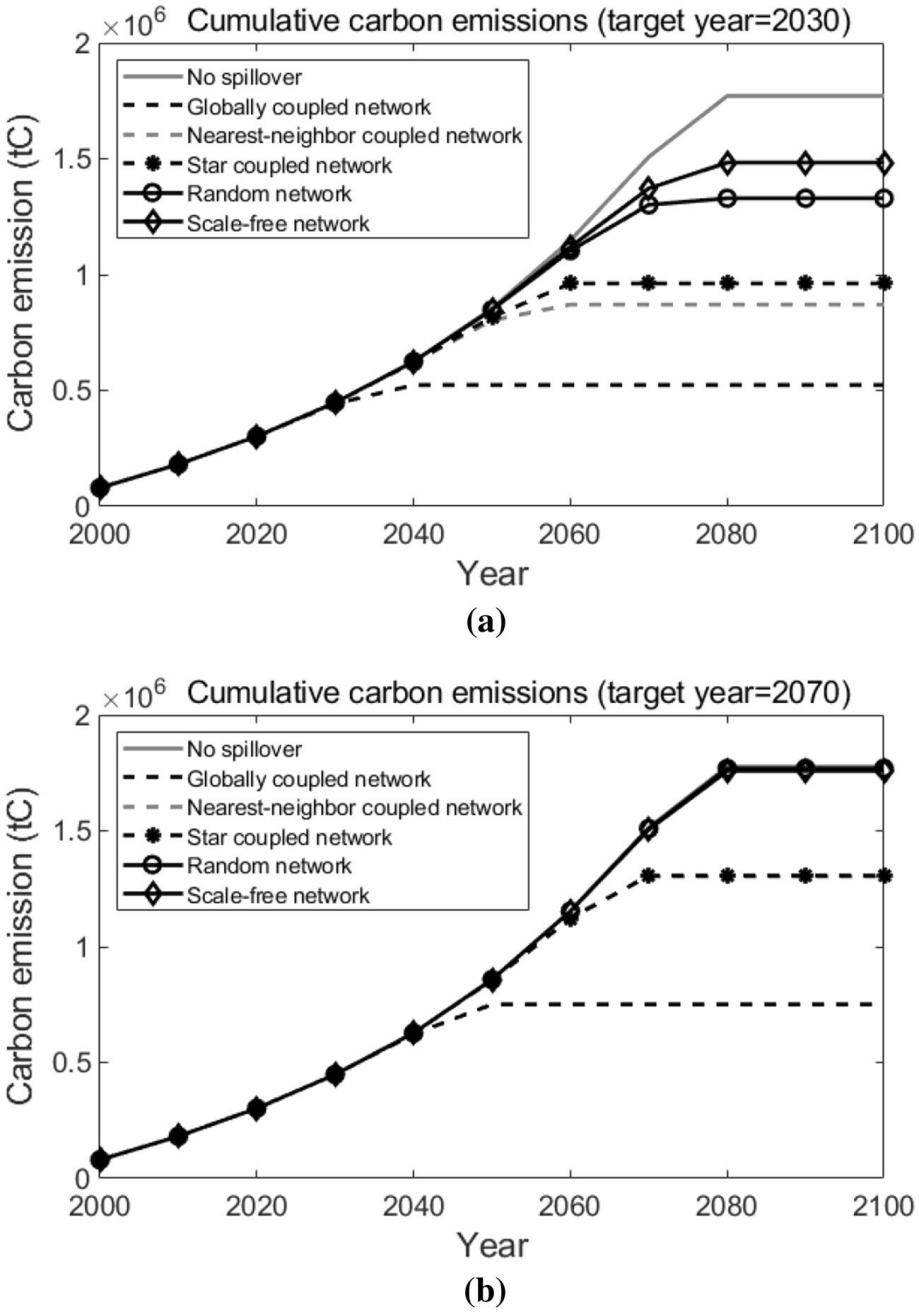
Sensitivity analysis on the target years. Sub-figure (**a**) presents the results when the target year = 2030, and sub-figure (**b**) presents the results when the target year = 2070.

- Learning rate of the new technology. A higher learning rate indicates a greater cost reduction potential for T3, and thus leads to the earlier adoption of T3. Simulation results are shown in Fig. 16. As we can observe, generally speaking, as the learning rate increases, the cumulative carbon emissions in all network topological structure situations decreases. When the learning rate decreases from the baseline (30%) to 27% or increases to 33%, over the 100 years, the spread between the highest cumulative carbon emission (which is in the scale-free network) and the lowest cumulative carbon emission (which is in the globally coupled network) is $1.48 \times 10^{6}$ tC and $6.37 \times 10^{5}$ tC, respectively. Compared with the results when the learning rate equals to the baseline (30%, as shown in Fig. 15b), we can conclude that, a higher learning rate leads to a smaller spread between the highest and the lowest cumulative carbon emissions under different network topological structures.
- The number of pilot agents. More pilot agents provide the system with more earlier adopters of the new advanced technology, which favors the diffusion of the new technology among agents. Simulation results are shown in Fig. 17. Compared with what suggested in Fig. 15b), when there are only 10 pilot agents in the entire system, we can see that a larger number of pilot agents leads to the lower cumulative carbon emissions in all network structure situations. When there are 100 or 500 pilot agents in the entire system, likewise, the lowest cumulative carbon emission over the decision horizon happens in the globally coupled network and the highest cumulative carbon emission happens in the scale-free network, and the spreads are $8.32 \times 10^{5}$ tC and $4.86 \times 10^{5}$ tC, respectively. It indicates that a larger number of pilot agents contributes to a lower spread in the cumulative carbon emissions when the network structure varies.
- The technological spillover rate. A comparatively larger value of the technological spillover rate indicates a lower technological barrier between the new technology’s earlier adopters and later adopters. Simulation results are shown in Fig. 18. As we can observe, when the technological spillover rate increases, the cumulative carbon emissions in all network topological structure situations decreases in general. When the spillover rate equals to 0.1 or 0.5, the lowest cumulative carbon emission also happens in the globally coupled network, but the highest cumulative carbon emission happens in the random network. The spreads between them over the 100 years are $7.60 \times 10^{5}$ tC and $4.02 \times 10^{5}$ tC, respectively. Together with the results suggested in Fig. 15b), we can conclude that, a larger spillover rate leads to a smaller spread of the cumulative carbon emissions under different network topological structures.
- The target year of the carbon emission constraints. An earlier target year indicates a more stringent carbon emission constraint for pilot agents, which requires them to complete the transition to the advanced clean technology earlier. Simulation results are shown in Fig. 19. As the target year postpones, the cumulative carbon emissions in all network topological structure situations increase on the whole. Similarly, the lowest cumulative carbon emission over the decision horizon happens in the globally coupled network. However, the highest cumulative carbon emissions over the decision horizon happen in the scale-free network when the target year advances to 2030 and in the random network when the target year postpones to 2070. The spreads between the highest and the lowest cumulative carbon emissions when the target year is 2030 and 2070 are $9.60 \times 10^{5}$ tC and $1.02 \times 10^{6}$ tC, respectively. Compared with the results in Fig. 15b) (when the target year is 2050), we can see that, the spread of the cumulative carbon emissions under different network structures changes within a small range when the target year of the carbon emission constraints changes.

The simulation results in “Discussion: how the network structure influences the effectiveness of a carbon emission policy intervention” imply that, although the real effectiveness of a carbon reduction policy intervention is largely determined by the network topological structure among the decision entities, there are several methods that could reduce the evaluation bias. For instance, set more pilot agents, or lower the technological barrier.

From Fig. 1 to Fig. 19, we can draw the following conclusions:

First, with different topological structures of the technological spillover network among agents, the later adopters commonly adopt T3 at different paces since they can only acquire new technology’s knowledge and experience from the earlier adopters through the channels determined by the network topological structures. As a result, T3’s adoption and diffusion in the entire system is also largely influenced by the network topological structures.

Second, the carbon emission constraints imposed on pilot agents can accelerate their adoption of the zero-emission new advanced technology. Due to technological spillover effect, other agents in the network could benefit from their knowledge and experience earlier. In this way, the carbon emissions of other agents in the system can also be reduced. Nonetheless, as we discussed before, the cumulative carbon emission reductions in the entire system due to the policy intervention are also at different levels under different network topological structures.

Third, the globally coupled network seems to be the most “optimistic” scenario, in which T3 is always adopted earliest and the cumulative carbon emission over the decision horizon is the lowest. When the technological spillover network is in the format of other topological structures, the cumulative carbon emissions of the entire system may not reduce as much as the globally coupled network shows, i.e., the policy intervention may not work as well as the most “optimistic” situation suggests. According to our simulation results, several factors could contribute to a smaller spread in the reduction of cumulative carbon emissions under different network topological structures, including a higher learning rate of the new advanced technology, a larger number of pilot agents, and a larger spillover rate.

## Conclusion and policy implications

This paper develops a systematic technology adoption model with technological spillover among agents from the network perspective. To investigate the role that topological structure of the technological spillover network plays, this paper first illustrates how the new technology diffuses from the leader agent to the follower agents, and how the new technology is adopted by individual agents and in the entire system under different network topological structures. Further, to illustrate the implication of our research, this paper examines how the carbon emission constraints imposed on pilot agents affect the carbon emissions of other agents and the entire system under different network topological structures.

Simulation results of our study suggest that, first, network topological structure has great influence on the agents’ decisions of adopting the new advanced technology. As a result, it also determines the new advanced technology’s development in the entire system largely; second, due to the technological spillover effect, carbon emission constraints imposed on pilot agents can also reduce the carbon emissions of other agents in the system. The carbon emission constraint policy seems to be most effective in the globally coupled network, since compared with other networks of different structures, its cumulative carbon emission in the entire system over the decision horizon is the lowest. Nonetheless, in the random network or in the scale-free network, the policy intervention may not function as effectively as the globally coupled network suggests. There’s always a spread between the highest cumulative carbon emission and the lowest cumulative carbon emission under different network topological structures. Our simulation results suggest that, several factors can contribute to reducing the spread, including a higher learning rate of the new advanced technology, a larger number of pilot agents, and a larger spillover rate.

Our simulations do not intend to find out which network topological structure has the most merits; instead, the purpose of our work is to bring cautions to social planners that different network topological structures affect the adoption and diffusion of a new advanced technology greatly, and also have large influence on the effectiveness of a carbon emission policy intervention. Therefore, the policy implication of our research is that, to evaluate the real effectiveness of a carbon emission reduction policy from the system level, the determination of the network topological structure among all the participating entities with real world data is a pre-requisite.

## Supplementary Information

Supplementary Information.
