# Supplementary material for: The impact of network topological structures on systematic technology adoption and carbon emission reduction
Source: Sci Rep. 2021 Oct 14;11:20380. doi: 10.1038/s41598-021-99835-3 (PMC8517025; doi:10.1038/s41598-021-99835-3)
Supplement: Supplementary file 1 — Supplementary Information. [file 41598_2021_99835_MOESM1_ESM.pdf]

The Impact of Network Topological Structures on Systematic Technology Adoption and Carbon Emission Reduction

Huayi Chen[1], Huai-Long Shi[2,*]

[1] College of Economics and Management, Nanjing University of Aeronautics and Astronautics, 29 Jiangjun Avenue, Nanjing 211106, China

[2] School of Management Science and Engineering, Nanjing University of Information Science and Technology, Nanjing 210044, China

[*] Corresponding author. E-mail: hlshi@nuist.edu.cn

Supplementary Methods

The systematic technology adoption model for each agent

Each decision agent minimizes the total cost over its decision-making period with the following objective function (S1):

$$min \ \sum_{i=1}^{3}\sum_{t=1}^{T}\frac{1}{(1+\delta)^t}CF_i^t y_i^t + \sum_{t=1}^{T}\frac{1}{(1+\delta)^t}C_E^t R^t + \sum_{i=1}^{3}\sum_{t=1}^{T}\frac{1}{(1+\delta)^t}C_{OMi}x_i^t, \qquad (S1)$$

where δ denotes the discount rate, and T denotes the length of one agent's decision-making period (i.e., its foresight).

The first part of the objective function represents the total investment cost of all three technologies, where, y_i^t denotes the newly installed capacity for technology *i* at time *t*; CF_i^t denotes the unit investment cost for technology *i* at time *t*, which for T1 is a constant. For T2 and T3, since we assume that they have learning potential, their unit investment costs are calculated with the following Eq. (S2):

$$CF_i^t = CF_i^0 \times \left(\bar{x}_i^{t-1}\right)^{-b_i}, \quad (i = 2{,}3) \qquad (S2)$$

where, CF_i^0 denotes the initial unit investment cost for technology *i*; $1-2^{-b_i}$ denotes the learning rate of technology *i*, which indicates the cost reduction percentage when the cumulative production of technology *i* doubles; b_i is the progress ratio; $\bar{x}_i^t$ represents the cumulative production of technology *i* at time *t*, which is calculated with the following Eq. (S3):

$$\bar{x}_i^t = \sum_{j=1}^{t} x_i^j + \bar{x}_i^0 \ , \qquad (S3)$$

where $\bar{x}_i^0$ denotes the initial cumulative production using technology *i* before the firstdecision period; x_i^j denotes the production using technology *i* at time *j*.

The second part of the objective function represents the total resource extraction cost,

where C_E^t denotes unit resource extraction cost at time *t*, which is computed with the following Eq. (S4):

$$C_E^t = C_E^0 + k_E \overline{R^t}, \tag{S4}$$

where C_E^0 denotes the initial unit resource extraction cost; k_E is a resource extraction cost coefficient; $\overline{R^t}$ denotes the cumulative resource extraction by time *t* :

$$\overline{R^t} = \sum_{j=1}^{t} R^j, \tag{S5}$$

where R^j is the total resource consumption at time *j*. It is the sum of the resources consumed by all three technologies at time *j* :

$$R^j = \sum_{i=1}^{3} \frac{1}{\eta_i} x_i^j, \tag{S6}$$

where η_i denotes the efficiency of technology *i*.

The third part of the objective function is the total operation and maintenance cost, where C_{OMi} denotes the unit OM cost for technology *i*.

The objective function is also subject to the following constraints

$$D^t \leq \sum_{i=1}^{3} x_i^t, \ (t = 1,2,\cdots,T) \tag{S7}$$

$$x_i^t \leq C_i^t, \ (t = 1,2,\cdots,T)(i = 1,2,3) \tag{S8}$$

$$x_i^t, y_i^t \geq 0, \quad (t = 1,2,\cdots,T)(i = 1,2,3) \tag{S9}$$

Inequation set (S7) indicates that the total production of all three technologies must satisfy its demand at each time *t*, where, D^t denotes the annual demand at time *t*, which increases over time with Eq. (S10):

$$D^t = D^0(1+\alpha)^t, \tag{S10}$$

where D^0 denotes the initial demand; α is a fixed annual growth rate and a constant.

Inequation set (S8) indicates that the production using one technology *i* cannot go beyond its total installed capacity at each time *t*. Here, C_i^t denotes the total installed capacity of technology *i* at time *t*, which is computed with the following Eq. (S11):

$$C_i^t = \begin{cases} \sum_{h=1}^{t} y_i^h + \frac{\tau_i - t}{\tau_i} C_i^0 & t \le \tau_i \\ \sum_{h=t-\tau_i}^{t} y_i^h & t > \tau_i \end{cases}, \tag{S11}$$

where, C_i^0 denotes the initial total installed capacity of technology *i* before the first decision period; τ_i is the plant life of technology *i*.

Inequation set (S9) denotes nonnegative constraints.

Table S1. Initial values of the parameters in the simulation

**Parameters**	**Existing technology (T1)**	**Incremental technology (T2)**	**Revolutionary technology (T3)**
Initial investment cost (US$/kW)	$CF_1^0 = 1{,}000$	$CF_2^0 = 2{,}000$	$CF_3^0 = 25{,}000$
Efficiency	$\eta_1 = 30\%$	$\eta_2 = 40\%$	$\eta_3 = 90\%$
Plant life (year)	$\tau_1 = 30$	$\tau_2 = 30$	$\tau_3 = 30$
Initial total installed capacity (kW)	$C_1^0 = 100$	$C_2^0 = 0$	$C_3^0 = 0$
Initial cumulative production (kWyr)	$\bar{x}_1^0 = 1$	$\bar{x}_2^0 = 1$	$\bar{x}_3^0 = 1$
OM cost (US$/kWyr)	$C_{OM1} = 30$	$C_{OM2} = 50$	$C_{OM3} = 50$
Learning rate	$1 - 2^{-b_1} = 0$	$1 - 2^{-b_2} = 10\%$	$1 - 2^{-b_3} = 30\%$
Emission coefficient (tC/kWyr)	$\lambda_1 = 0.8$	$\lambda_2 = 0.64$	$\lambda_3 = 0$

**Other parameters**	
Initial demand (kWyr)	$D^0 = 100$
Annual growth rate of demand	$\alpha = 2.6\%$
Initial resource extraction cost (US$/kWyr)	$C_E^0 = 200$
Resource extraction cost coefficient	$k_E = 0.01$
Discount rate	$\delta = 5\%$
Spillover rate	$\theta = 1\%$
